# [^18^F] AlF‑NOTA‑FAPI‑04 PET/CT as a promising tool for imaging fibroblast activation protein in gastrointestinal system cancers: a prospective investigation of comparative analysis with ^18^F-FDG

**DOI:** 10.1007/s00259-023-06351-9

**Published:** 2023-08-05

**Authors:** Liping Yang, Shichuan Xu, Liang Cheng, Chao Gao, Shaodong Cao, Zhengsong Chang, Kezheng Wang

**Affiliations:** 1 Department of PET-CT, Harbin Medical University Cancer Hospital, Harbin, China; 2 Department of Medical Instruments, Second Hospital of Harbin, Harbin, China; 3 Department of Medical Imaging, The Fourth Affiliated Hospital of Harbin Medical University, Harbin, China; 4 Department of Pathology, Harbin Medical University Cancer Hospital, Harbin, China

**Keywords:** Gastrointestinal system cancers, FAPI PET/CT, FDG PET/CT, Cancer-associated fibroblast, Clinical management

## Abstract

**Purpose:**

The radiopharmaceutical [^18^F]AlF-NOTA-FAPI-04 presents a promising alternative to ^68^ Ga-FAPI owing to its relatively longer half-life. This study aimed to evaluate the clinical usefulness of [^18^F]AlF-NOTA-FAPI-04 PET/CT for the diagnosis of primary and metastatic lesions in various types of gastrointestinal system cancers, compared with ^18^F-FDG PET/CT.

**Methods:**

Patients diagnosed with gastrointestinal system malignancies were prospectively enrolled. All patients underwent both ^18^F-FDG and ^18^F-FAPI-04 PET/CT scans within one week, with 44 (73.3%) for cancer staging and 16 (26.7%) for tumor restaging. Diagnostic efficacy of the primary tumor, as well as the presence and number of lymph nodes and distant metastases, were assessed. Tumor uptake was quantified by the maximum standard uptake value (SUVmax).

**Results:**

For detection of primary tumor, the diagnostic sensitivity of ^18^F-FDG PET/CT was 72.7%, while it was 97.7% for ^18^F-FAPI-04 PET/CT. Based on per-lymph node analysis, the sensitivity, specificity, and accuracy of ^18^F-FAPI-04 PET/CT in diagnosing metastatic lymph nodes were 91.89%, 92.00%, and 91.96%, respectively. These values were notably higher than those ^18^F-FDG PET/CT (79.72%, 81.33% and 80.80%, respectively). The ^18^F-FAPI-04 PET/CT surpassed ^18^F-FDG PET/CT in detecting suspected metastases in the brain (7 vs. 3), liver (39 vs. 20), bone (79 vs. 51), lung (11 vs. 4), and peritoneal carcinoma (48 vs. 22). Based on per-patient analysis, differential diagnostic accuracies (^18^F-FAPI-04 vs. ^18^F-FDG PET/CT) were observed in all patients (91.7% vs. 76.7%), the initial staging group (90.9% vs. 79.5%), and the re-staging group (93.8% vs. 68.7%). Additionally, ^18^F-FAPI-04 PET/CT revised final diagnosis in 31.7% of patients, contrasting with ^18^F-FDG PET/CT, and prompted changes in clinical management for 21.7% of the patients.

**Conclusion:**

^18^F-FAPI-04 PET/CT outperforms ^18^F-FDG PET/CT in delineating the primary gastrointestinal tumors and detecting suspected metastatic lesions due to a higher target-to-background ratio (TBR). Moreover, ^18^F-FAPI-04 PET/CT could provide valuable guidance for tumor staging, thereby having a potential impact on patient management.

**Supplementary Information:**

The online version contains supplementary material available at 10.1007/s00259-023-06351-9.

## Introduction

Alterations in metabolism are among the hallmarks of malignant tumors. The transition to increased glucose metabolism, characteristic of cancerous cells, was first observed since the 1980s through fluorine-18 fluorodeoxyglucose (^18^F-FDG) PET/CT imaging [1]. Consequently, ^18^F-FDG uptake is linked to glucose metabolism levels and is frequently employed as a diagnostic radiotracer for oncological PET imaging. Despite consensus on the significant contributions of ^18^F-FDG PET/CT to tumor staging, therapeutic efficacy assessment, and recurrence monitoring in gastrointestinal system cancers, its limitations cannot be overlooked [2]. A decreased sensitivity in detecting early-stage or specific subtypes of gastrointestinal system malignancies has been reported, attributed to the slow proliferation of these tumor cells. In addition, the ability of ^18^F-FDG PET/CT to detect regional lymph node metastasis is suboptimal, with a sensitivity of only 55%, leading to subpar treatment and poor survival outcomes [3]. Thus, there is an urgent need to develop an effective PET radiotracer to facilitate accurate tumor characterization and personalized patient management.

It's well-established that the tumor microenvironment has an indispensable role in fostering neoplasia development. Cancer-associated fibroblasts (CAFs) are the dominant components of the tumor microenvironment. Research has shown that CAFs are critical catalysts for tumor growth, invasion, metastatic spread, and they're closely linked with treatment resistance and poor survival prognosis [4]. Fibroblast activation protein (FAP), a type II transmembrane serine protease, is scarcely found in normal tissues and organs, but is overexpressed in CAFs in various epithelial carcinomas. CAFs enable promote tumor cell migration, invasion, angiogenesis, and metastasis by activating corresponding signaling pathways [5]. Given these properties, Gallium-68-labeled fibroblast activation protein inhibitor (^68^Ga-FAPI) has emerged as a novel FAP-targeting radiotracer for PET cancer imaging, promising in vivo visualization of tumor stroma. Among these FAPIs, FAPI-04 stands out due to its enhanced FAP binding capacity and favorable pharmacokinetics, making it ideal for contrast and visibility [6]. This led to the development of ^68^Ga-DOTA-FAPI-04 PET/CT for fast imaging of a wide range of tumors.

Current research on molecular imaging probes targeting FAP commonly uses ^68^Ga-FAPI-04 for PET imaging. Despite the unprecedented success of ^68^Ga-FAPI-04 PET/CT in detecting primary tumors, it has its drawbacks. The broad application of ^68^Ga-labeled FAPI in clinical practice is limited due to the short half-life of ^68^Ga, high costs, and insufficient availability of radionuclides from the ^68^Ge/^68^Ga generator. Conversely, ^18^F is the most widely used radionuclide in PET imaging, as it can be mass-produced via a cyclotron and transported over long distances [7]. Consequently, ^18^F-FAPI-04 emerges as an ideal alternative to ^68^Ga-FAPI-04. Preclinical evaluations of ^18^F-FAPI-04 PET/CT have demonstrated promising results in cancer imaging of FAP expression in mice [8], proving its safety and feasibility for further clinical translation. However, a paucity of studies directly comparing these two PET radiotracers (^18^F-FAPI-04 and ^18^F-FDG) in characterizing primary tumors and metastatic lesions can be noted in current literature. Therefore, our study aims to conduct a prospective, head-to-head comparison of ^18^F-FAPI-04 to ^18^F-FDG in patients with various gastrointestinal system cancers to establish generalizable differences between these two agents.

## Materials and methods

### Study population

This prospective study was reviewed, approved, and overseen by the institutional review board of Harbin Medical University Cancer Hospital (approval 2021-198-JS) and conducted under the 1964 Declaration of Helsinki and its later amendments or comparable ethical standards. The study was registered at ClinicalTrials.gov (ChiCTR2200058108). All subjects signed informed consent forms in line with the local ethics committee's regulations for prospective research. ^18^F-FAPI-04 PET/CT was performed for comparative purposes within one week after ^18^F-FDG PET/CT. Participants were consecutively recruited for enrollment in this study from October 2021 to December 2022.

The specific inclusion criteria were as follows: 1) adult patients (≥ 18 years old); 2) subjects with newly diagnosed or previously treated gastrointestinal system malignancies (The interval between the completion of anti-tumor treatment and the PET-CT scan was more than 6 months); 3) patients who agreed to undergo paired ^18^F-FDG and ^18^F-FAPI-04 PET/CT for tumor staging or restaging; 4) according to RECIST1.1, there was at least one measurable target lesion; 5) understand and sign informed consent voluntarily with good compliance. Exclusion criteria included 1) the function of liver and kidney was seriously abnormal; 2) preparation for pregnant, pregnant and lactating women; 3) patients whose treatment had already started between the acquisition of the ^18^F-FDG PET/CT scan and ^18^F-FAPI-04 PET/CT scan; 4) inability to lie flat for half an hour; 5) suffering from claustrophobia or other mental disorders; 6) other researchers considered it unsuitable to participate in the trial; 7) substandard image quality (such as motion artifacts).

All patients were classified into either an initial staging group or a restaging group. The former refers to those patients who had not received any treatment before examinations. The latter was defined as patients who underwent examinations during treatment (therapeutic effect evaluation) or at least 2 months after completion of treatment (Monitoring tumor recurrence or metastasis). In this study, histopathologic examination of a biopsy or resected surgical specimens served as the gold standard for the final diagnosis. Clinical follow-up information, including results of medical imaging, physical examination, and laboratory tests, was used as the final reference standard when the pathological diagnosis is unavailable. All patients have to receive at least a three-month follow-up period.

### Synthesis of ^18^F-FDG and ^18^F-FAPI-04

^18^F-FDG was automatically manufactured at the PET/CT department of Harbin Medical University Cancer Hospital by the standard preparation methods applying the coincidence ^18^F-FDG synthesis module (TracerLab Fx FDG; GE Healthcare, Milwaukee, Wis). Radiolabeling of ^18^F-FAPI-04 was performed using a previously described protocol. The FAPI precursor (1,4,7-triazacyclononane-1,4,7-triacetic acid) [NOTA] FAPI-04). Quality control of the radiosynthesis was performed by ultraviolet and radio high-performance liquid chromatography (HPLC). Radiochemical purity exceeded 95% for both ^18^F-FAPI-04 and ^18^F-FDG, and the final product was diluted and sterilized. The sterility tests were conducted in the radiochemistry facility of Harbin Medical University Cancer Hospital. Finally, ^18^F-FDG and ^18^F-FAPI-04 have to conform to all set criteria prior to their transformation into the clinic for human administration. The synthesis process of ^18^F‑FAPI‑04 is described in supplementary data 1, and the corresponding chemical structural formula is displayed in supplementary data 2.

### Image acquisition

^18^F-FAPI-04 PET/CT examination should be performed within one week after ^18^F-FDG PET/CT scanning. The intravenous dose of the two agents is calculated according to the patient's weight (3.7 MBq [0.1 mCi]/kg for FDG; 1.8–2.2 MBq [0.05–0.06 mCi]/kg for FAPI). Before the ^18^F-FDG PET/CT examination, each patient was required to fast for 4-6 hours, to achieve blood glucose levels < 160 mg/dl. All patients do not need special preparation (e.g., fasting and normal blood glucose level) before the ^18^F-FAPI-04 PET/CT scans. Static PET/CT imaging was performed using a hybrid PET/CT system (Discovery MI, GE Healthcare, Milwaukee, WI, USA) 60 min after injection. Firstly, low-dose CT scans (free-breathing state and unenhanced images) were performed before whole-body PET/CT examination. Detailed scanning parameters were as follows: tube voltage 140 kV, tube current 150 mA, slice thickness 3.75 mm, matrix size 512 × 512, and field of view 450 mm. PET examination was immediately performed after the CT examination in three-dimensional acquisition mode with 6–8 bed positions and 2.0–2.5 min/position. Image reconstructions were performed based on the 3D ordered subset expectation-maximization algorithm (2 iterations and 17 subsets), and all PET images were corrected for attenuation correction and reconstructed into a 128 × 128 matrix.

### Image analysis and clinical staging

Images were analyzed using a dedicated post-processing software (PET VCAR; GE Healthcare). Two experienced radiologists who have more than 10-year practicing experience, blinded to the surgical and pathological results, independently evaluated the images. Any discrepancy was settled through consensus by discussion. To prevent any bias, ^18^F-FDG PET/CT images were evaluated by group 1 (radiologist S.X. and radiologist C.G.), while ^18^F-FAPI-04 PET/CT images were assessed in group 2 (radiologist K.W. and radiologist S.C.). All of them were blinded to patient history as well as results of conventional imaging and pathologic results. For visual analysis, based on the understanding of the physiological distribution of the agents in the whole body, compared with the contralateral normal tissues and surrounding soft tissues, if radiotracer uptake is increased or significantly concentrated, which exceeded the average value of adjacent background tissues, then these lesions were considered as positive. For semiquantitative analysis, regions of interest were manually drawn by another radiologist (L.C., with more than 10 years of experience in nuclear oncology) on trans-axial images around the tumor lesions to measure the metabolic parameters. The maximum standard uptake values (SUVmax), the median, and range of standard uptake values were recorded, which were used to quantify the radiotracer uptake in primary tumors, lymph nodes, and metastatic lesions. The tumor-to-background (T/B) ratio was recorded by dividing the tumor SUVmax with the mean SUV of the contralateral normal tissue.

For tumor initial staging, the eighth edition of the AJCC TNM staging system was applied [9]. In brief, the degree of primary tumor invasion defines its T-stage, the number and distribution of metastatic lymph nodes define its N-stage, and the situation of distant metastasis determines its M-stage. For tumor restaging, suspected local recurrence was determined as the occurrence of new lesions at the primary tumor site, and metastasis was defined as the presence of metastatic lymph nodes or/and distant metastases. Lymph nodes mainly involved four regions: neck and supraclavicular, mediastinum, abdomen (including paraaortic, porta hepatic, retroperitoneal, celiac), and pelvis. Any peritoneal or omental or mesenteric metastasis is considered peritoneal carcinoma. Each metastasis of the brain, bone, liver, lung, and peritoneal carcinoma was uniformly defined as distant metastases.

For per-patient analysis, A final diagnosis was made by a dedicated multidisciplinary team (MDT) based on a comprehensive evaluation of clinical symptoms and signs, laboratory tests, radiological findings, pathological results, and clinical follow-up outcome. The MDT is composed of the previously mentioned nuclear medicine experts, two tumor surgery experts, one medical oncologist, and one radiation oncologist, who finally reached a consensus on the final diagnosis as the reference standard. Based on the difference between the two imaging methods and the reference standard in the same patient, final results were classified as the same (correct), overestimated, or underestimated. According to the above findings, we recorded the changes in clinical staging, and subsequent changes in oncological management were assessed by two nuclear medicine experts and two treating physicians. The referring treating physicians have further been consulted on what the therapeutic regimen would be prior to and after ^18^F-FAPI-04 PET/CT. The management reference standard was the consensus of the above-mentioned MDT team in line with the final diagnosis and the latest NCCN guidelines. For patients in the initial staging group, the therapeutic regimen included no treatment needed/follow-up, surgical resection, perioperative chemotherapy/radio-chemotherapy plus surgical resection, and non-surgical candidate. For those patients in the re-staging group, the secondary treatment plan was compared with the previous ones and then classified as maintenance or modification treatment.

### Statistical analysis

Statistical analysis was performed using SPSS software (version 23.0, Chicago, IL, USA). The continuous variables were summarized with means ± standard deviations and the categorical variables are denoted as numbers and percentages. The Wilcoxon matched-pairs signed-rank test was applied to compare the number of detected positive lesions based on the two imaging modalities. The value of metabolic parameters measured on different imaging methods was also compared using the same test. *P* value with two-side below 0.05 was considered statistically significant.

## Results

### Baseline demographics

Between October 2021 and December 2022, sixty-four patients with gastrointestinal system cancers were studied, who underwent both ^18^F-FDG and ^18^F-FAPI-04 PET/CT scans. Among the finally included 60 patients, 34 patients were female and 26 were males, and the medial age of the patients was 61 years (range, 38–68 years). The characteristics of the patients are summarized in Table 1. The median between ^18^F-FDG and ^18^F-FAPI-04 PET/CT was 2 days (range 1–5) days. The mean clinical follow-up time was 5.2 ± 1.8 month.

**Table 1:** Patient characteristics

Description of patients (*n* = 60)	Value
Age (years)	
Median	61
Interquartile range	38–68
Gender, No. (%)	
Female	34 (56.7%)
Male	26 (43.3%)
Indication for PET, No. (%)	
Initial staging	44 (73.3%)
Restaging	16 (26.7%)
Tumor types, No. (%)	
Gastric cancer	18 (30.0%)
Pancreatic cancer	16 (26.7%)
Liver cancer	12 (20.0%)
Colorectal cancer	14 (23.3%)
Patient status, No. (%)	
Treatment-naive	44 (73.3%)
Surgery	5 (8.3%)
Chemotherapy, and radiotherapy	2 (3.4%)
Surgery and chemotherapy	3 (5.0%)
Surgery, chemotherapy, and radiotherapy	6 (10.0%)
Interval between two examinations (days)	
Median	2
Interquartile range	1–5
Follow-up period (months), mean ± SD	5.2 ± 1.8

*SD* Standard Deviation

Among these newly diagnosed patients, fourteen patients had gastric cancer, including 12 patients with adenocarcinoma and 2 patients with signet ring cell carcinoma, ten patients had liver cancer, including 8 patients with hepatocellular carcinoma and 2 patients with intrahepatic cholangiocarcinoma, twelve patients had pancreatic cancer, including 3 patients with well-differentiated adenocarcinoma, 5 patients with moderately-differentiated adenocarcinoma and 4 patients with poorly-differentiated adenocarcinoma, eight patients had colorectal cancer, including 6 patients with moderately-differentiated adenocarcinoma and 2 patients with poorly-differentiated adenocarcinoma. All individuals who had previous treatment for gastrointestinal system cancers, in which four instances were gastric cancer (3 patients with adenocarcinoma and 1 patient with signet ring cell carcinoma), two instances were liver cancer (1 patients with hepatocellular carcinoma and 1 patient with intrahepatic cholangiocarcinoma), four instances were pancreatic ductal adenocarcinoma and six instances were colorectal adenocarcinoma.

### Experimental safety

No drug-related adverse reactions occurred before or after ^18^F-FAPI-04 PET/CT scans. PET imaging was tolerated well by all patients and any abnormal symptoms were not observed during injection and the 2 hours of observation period.

### Detection of primary tumors

A total of 44 primary lesions were examined in patients with newly diagnosed gastrointestinal system cancers. In the depiction of primary tumors, the detection rate was 72.7% (32 of 44) for ^18^F-FDG PET/CT and 97.7% (43 of 44) for ^18^F-FAPI-04 PET/CT. The false-negative tumors from ^18^F-FDG PET/CT were pancreatic cancer (n = 3), gastric cancer (n = 4), and liver cancer (n = 5). ^18^F-FAPI-04 PET/CT demonstrated a higher detection rate for primary tumors in the initial staging group (97.7% [43 of 44] vs. 72.7% [32 of 44], *P* = 0.032). Only one primary lesion from the pancreatic was not detected on ^18^F-FAPI-04 PET/CT images, which was covered by diffuse and intense tracer uptake in the whole pancreas. On the ^18^F-FAPI-04 PET/CT images, most primary tumors were delineated with clear tumor contour and demonstrated a higher TBR (7.2 vs. 2.4; *P* < 0.001) than ^18^F-FDG, particularly in patients with gastric cancer and liver cancer. For semiquantitative analysis (Table 2), a higher uptake of ^18^F-FAPI-04 in pancreatic cancer compared with ^18^F-FDG (median SUV max, 10.4 vs. 5.1, respectively; *P* < 0.001), in gastric cancer (median SUV max, 9.7 vs. 4.6, respectively; *P* < 0.001), in liver cancer (median SUV max, 11.2 vs. 4.2, respectively; *P* < 0.001). Although all primary lesions from colorectal tumors were visualized on ^18^F-FDG PET/CT, these primary tumors demonstrated a higher uptake of ^18^F-FAPI-04 than of ^18^F-FDG (median SUV max, 9.1 vs. 5.7, respectively; *P* < 0.001).

**Table 2:** Comparison of ^18^F-FDG and ^18^F-FAPI-04 uptake in primary tumor, lymph nodes and distant metastases

	^18^F-FDG		^18^F-FAPI-04		*P* value
Parameters	Median SUVmax	Range of SUVmax	Median SUVmax	Range of SUVmax	(SUVmax-FAPI *vs.* SUVmax-FDG*)*
Primary tumor					
Gastric cancer	4.6	1.9–10.8	9.7	4.8–16.9	< 0.001
Pancreatic cancer	5.1	3.4–11.2	10.4	5.4–20.6	< 0.001
Liver cancer	4.2	2.0–10.4	11.2	7.6–21.3	< 0.001
Colorectal cancer	5.7	2.3–12.5	9.1	4.2–17.4	< 0.001
Involved lymph nodes					
Neck and supraclavicular	2.9	1.6–7.4	5.2	4.3–7.9	< 0.001
Mediastinum	4.9	1.8–10.5	5.4	4.8–14.7	0.382
Abdomen	3.7	2.0–14.2	6.6	5.2–16.2	< 0.001
Pelvis	3.3	1.6–11.7	6.0	4.5–13.8	< 0.001
Distant metastases					
Bone	3.1	1.7–11.5	5.2	3.4–13.1	< 0.001
Liver	4.4	1.9–8.9	8.7	4.2–18.7	< 0.001
Lung	2.7	1.4–6.2	4.5	2.7–6.9	< 0.001
Brain	3.2	1.6–3.9	3.8	1.9–4.3	0.247
Peritoneal carcinoma	3.6	2.1–12.7	8.1	6.2–19.4	< 0.001

Lymph nodes in abdominal regions includes paraaortic, porta hepatic, retroperitoneal and celiac lymph nodes. Any peritoneal or omental or mesenteric metastasis is considered as peritoneal carcinoma

### Detection of nodal metastasis

The number of positive lymph nodes detected with ^18^F-FAPI-04 PET/CT in the abdomen (154 vs. 69) and pelvis (38 vs. 20) regions was more than that detected with ^18^F-FDG PET/CT, and the uptake of ^18^F-FAPI-04 was higher than that of ^18^F-FDG (median SUV max, 6.6 vs. 3.7, *P* < 0.001; 6.0 vs. 3.3, *P* < 0.001, respectively,). The positive lymph nodes in the region of the neck and supraclavicular demonstrated no difference in the number detected on ^18^F-FAPI-04 and ^18^F-FDG PET/CT (28 vs. 23), whereas the uptake of ^18^F-FAPI-04 was higher than that of ^18^F-FDG (median SUV max, 5.2 vs. 2.9, respectively; *P* < 0.001). Lymph nodes in the region of the mediastinum demonstrated no difference in uptake of radiotracers between ^18^F-FAPI-04 and ^18^F-FDG (median SUV max, 5.4 vs. 4.9, respectively; *P*= 0.382), whereas ^18^F-FAPI-04 PET/CT surpassed ^18^F-FDG PET/CT in the detection of mediastinal lymph nodes (19 vs. 9). A sum of 224 suspicious lymph nodes was identified in 36 patients and validated via lymph node dissection (n = 182), biopsy (n = 5), or radiographic follow-up (n = 37). Of these, metastasis was validated in 74 lymph nodes in 33 patients. ^18^F-FAPI-04 PET/CT depicted involved lymph nodes with 68 true-positive, 12 false-positive, 6 false-negative, and 138 true-negative. By contrast, ^18^F-FDG PET/CT depicted involved lymph nodes with 59 true-positive, 12 false-positive, 15 false-negative, and 122 true-negative. In per-lymph node analysis, the sensitivity, specificity, and accuracy in the diagnosis of metastatic lymph nodes were 91.89% (68 of 74), 92.00% (138 of 150), and 91.96% (206 of 224), respectively, for ^18^F-FAPI-04 PET/CT and 79.72% (59 of 74), 81.33% (122 of 150), and 80.80% (181 of 224) for ^18^F-FDG PET/CT. Both the sensitivity and the specificity of ^18^F-FAPI-04 PET/CT outperformed that of ^18^F-FDG PET/CT (91.89% vs. 79.72%, 92.00% vs. 81.33%, respectively; both *P* values < 0.001). The diagnostic performance in lymph node metastasis based on ^18^F-FAPI-04 and ^18^F-FDG PET/CT is displayed in Table 3.

**Table 3:** Diagnostic performance of ^18^F-FAPI-04 and ^18^F-FDG PET-CT

	^18^F-FDG			^18^F-FAPI-04		
Parameters	Sensitivity (%)	Specificity (%)	Accuracy (%)	Sensitivity (%)	Specificity (%)	Accuracy (%)
Lymph node metastases	79.72% (59/74)	81.33% (122/150)	80.80% (181/224)	91.89% (68/74)	92.00% (138/150)	91.96% (206/224)
Distant metastases	72.34% (102/141)	68.00% (17/25)	73.46% (119/162)	91.24% (125/137)	84.00% (21/25)	73.46% (119/162)

### Detection of distant metastasis

Each lesion of the brain, liver, bone, lung, and peritoneal carcinoma was recorded separately. Compared with ^18^F-FDG PET/CT, ^18^F-FAPI-04 PET/CT depicted more suspected metastases in brain (7 vs. 3), liver (39 vs. 20), bone (79 vs. 51), lung (11 vs. 4) and, peritoneal carcinoma (48 vs. 22). For semiquantitative analysis, the median SUV max values derived from ^18^F-FAPI-04 were higher than that from ^18^F-FDG in most of the bone (5.2 vs. 3.1, respectively; *P* < 0.001), liver metastases (8.7 vs. 4.4, respectively; *P* < 0.001), lung metastases (4.5 vs. 2.7, respectively; *P* < 0.001) and peritoneal carcinoma (8.1 vs. 3.6, respectively; *P* < 0.001). It is also worth noting that lesions of brain metastases showed no significant difference in uptake of radiotracers between ^18^F-FAPI-04 and ^18^F-FDG (median SUV max, 3.8 vs. 3.2, respectively; *P*= 0.247), whereas the TBRs on ^18^F-FAPI-04 PET/CT surpassed that on ^18^F-FDG PET/CT (median, 9.6 vs. 2.5).

Regarding diagnostic performance for distant metastases, we evaluated 162 suspicious lesions in 40 patients. Pathological findings via surgery (n = 59), biopsy (n = 45), or radiographic follow-up (n = 58) were used to evaluate suspicious lesions. Of these, 137 lesions were confirmed as positive metastases in 38 patients. ^18^F-FAPI-04 PET/CT depicted involved distant metastatic lesions with 125 true-positive, 4 false-positive, 12 false-negative, and 21 true-negative. By contrast, ^18^F-FDG PET/CT depicted involved distant metastatic lesions with 102 true-positive, 8 false-positive, 35 false-negative, and 17 true-negative. However, it is also worth noting that biopsy validation of all suspicious distant metastatic lesions in this study was only used to verify PET/CT findings. Therefore, the true-negative and false-positive status of these patients cannot be accurately determined. In per-distant metastases analysis, the sensitivity, specificity, and accuracy in the diagnosis of distant metastatic lesions were 91.24% (125 of 137), 84.00% (21 of 25), and 73.46% (119 of 162), respectively, for ^18^F-FAPI-04 PET/CT and 72.34% (102 of 141), 68.00% (17 of 25), and 73.46% (119 of 162) for ^18^F-FDG PET/CT. Both the sensitivity and the specificity of ^18^F-FAPI-04 PET/CT were superior to that of ^18^F-FDG PET/CT (91.24% vs. 72.34%, 84.00% vs. 68.00%, respectively; both *P* values < 0.001). The summary of diagnostic performance in distant metastatic lesions based on ^18^F-FAPI-04 and ^18^F-FDG PET/CT is displayed in Table 3.

### Diagnostic accuracy and clinical staging

On patient-based analysis, differential diagnostic accuracies (^18^F-FAPI-04 vs. ^18^F-FDG PET/CT) were observed in all patients (91.7% vs. 76.7%), the initial staging group (90.9% vs. 79.5%), and the re-staging group (93.8% vs. 68.7%), all *P* values were less than 0.001. Among these misdiagnosed patients, 80.0% (4/5) were overestimated on ^18^F-FAPI-04 PET/CT and 100 % (14/14) were underestimated on ^18^F-FDG PET/CT (Table 4). Regarding the diagnostic consistency of ^18^F-FAPI-04 and ^18^F-FDG PET/CT, ^18^F-FAPI-04 PET/CT amended the underestimation of ^18^F-FDG PET/CT in 19 patients (31.7%, 19/60), including 10 patients in the initial staging group and 9 patients in the restaging group (Table 5). As demonstrated in Table 6, the overall consistency of the oncological management recommended by ^18^F-FAPI-04 PET/CT with the reference standard was much higher than that of ^18^F-FDG PET/CT in the re-staging group. However, it is worth noting that the differences were not significant (*P*=0.098) in the initial staging group. Similarly, regarding the management consistency recommended by ^18^F-FAPI-04 and ^18^F-FDG PET/CT, among the patients who would have been misdirected according to ^18^F-FDG PET/CT, the treatment plans of 13 were corrected by ^18^F-FAPI-04 PET/CT (7 in the initial staging group and 6 in the restaging group), which means that ^18^F-FAPI-04 PET/CT prompted management changes in 13/60 (21.7%) patients (see details in supplementary data 3).

**Table 4:** Diagnostic accuracy and number of misdiagnoses of ^18^F-FDG and ^18^F-FAPI-04 PET-CT

	^18^F-FAPI-04	^18^F-FDG	FAPI *vs.* FDG
Overall (no.)	60	60	(*P*-value)
Accuracy	91.7%	76.7%	< 0.001
Number of misdiagnoses	5	14	
Overestimated	4	0	
Underestimated	1	14	
Initial staging group (no.)	44	44	
Accuracy	90.9%	79.5%	< 0.001
Number of misdiagnoses	4	9	
Overestimated	3	0	
Underestimated	1	9	
Restaging group (no.)	16	11	
Accuracy	93.8%	68.7%	< 0.001
Number of misdiagnoses	1	5	
Overestimated	1	0	
Underestimated	0	5

**Table 5:** Diagnostic consistency of ^18^F-FDG and ^18^F-FAPI-04 PET-CT

	Overall	Initial staging group	Restaging group
	FAPI and FDG	FAPI and FDG	FAPI and FDG
No	60	44	16
Consistent	38	32	6
Both correct	35	30	5
Both wrong	3	2	1
Overestimated	0	0	0
Underestimated	0	0	0
Inconsistent	22	12	10
FAPI correct and FDG wrong	19	10	9
FDG overestimated	0	0	0
FDG underestimated	19	10	9
FAPI wrong and FDG correct	0	0	0
FAPI overestimated	0	0	0
FAPI underestimated	0	0	0
Both wrong	3	2	1
FAPI overestimated	3	2	1
FAPI underestimated	0	0	0
FDG underestimated	3	2	1

**Table 6:** Comparison of management consistency of ^18^F-FDG and ^18^F-FAPI-04 PET-CT with the reference management

	^18^F-FAPI-04	^18^F-FDG	FAPI *vs.* FDG
			(*P*-value)
Overall	91.6% (56/60)	78.3% (47/60)	< 0.010
Initial staging group	93.1% (41/44)	84.1% (37/44)	0.098
Restaging group	87.5% (14/16)	62.5% (10/16)	< 0.001

### Representative cases

**Fig. 1:**
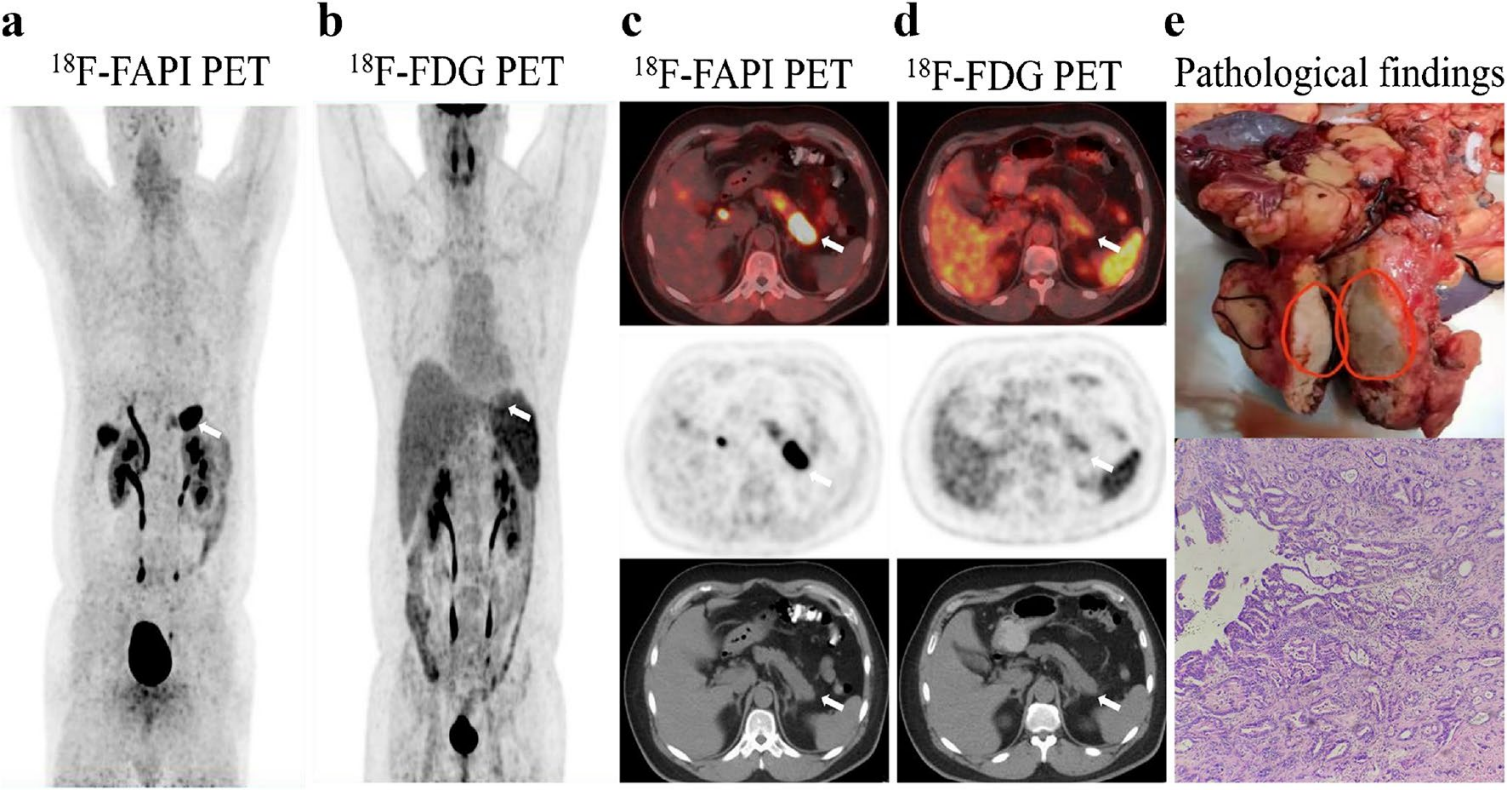
A 43-year-old male underwent PET/CT to evaluate a pancreatic mass previously detected through ultrasound. **a**, **c** ^18^F-FAPI-04 PET/CT images show one extremely strong uptake lesion (SUVmax = 25.3) in the tail of pancreas (arrows), which were suspected of pancreatic cancer. **b**, **d** ^18^F-FDG PET/CT images show mild tracer uptake (SUVmax = 5.50) in the corresponding pancreatic lesion (arrows). Compared with ^18^F-FDG PET/CT, ^18^F-FAPI-04 PET/CT demonstrate higher TBR (19.5 vs. 2.15) and clearer tumor contour. **e** The pathological results derived from a pancreatic mass resection revealed a primary moderately differentiated ductal adenocarcinoma of the pancreas

**Fig. 2:**
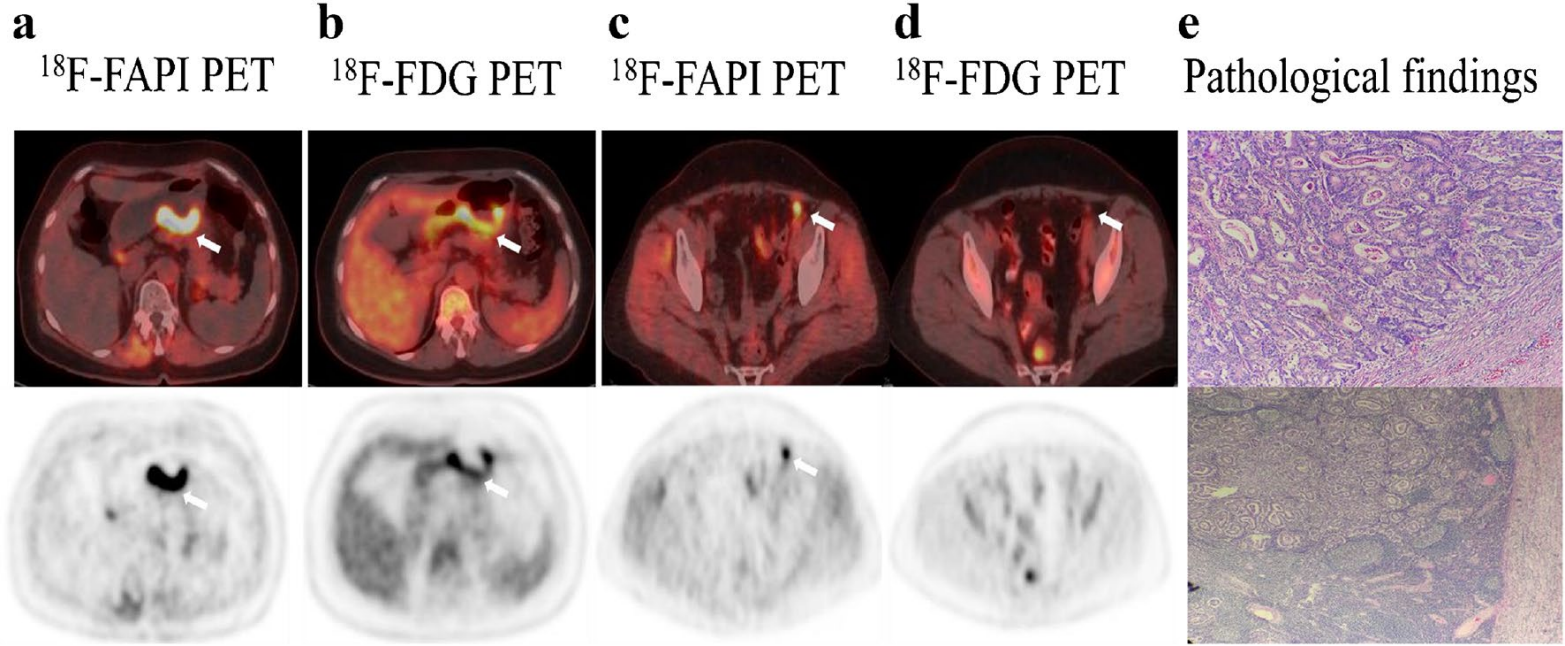
A 67-year-old female patient with gastric adenocarcinoma and regional lymph node metastasis postoperatively confirmed by pathology. **a**, **c** ^18^F-FAPI-04 PET/CT displayed diffusely strong uptake in the primary lesion (SUVmax = 9.7) and lymph node (SUVmax = 3.3) (arrows). **b**, **d** ^18^F-FDG PET/CT showed moderate uptake in the primary tumor (SUV max = 6.9) but no uptake in the regional lymph node. **e** The pathological results validated our radiological findings

**Fig. 3:**
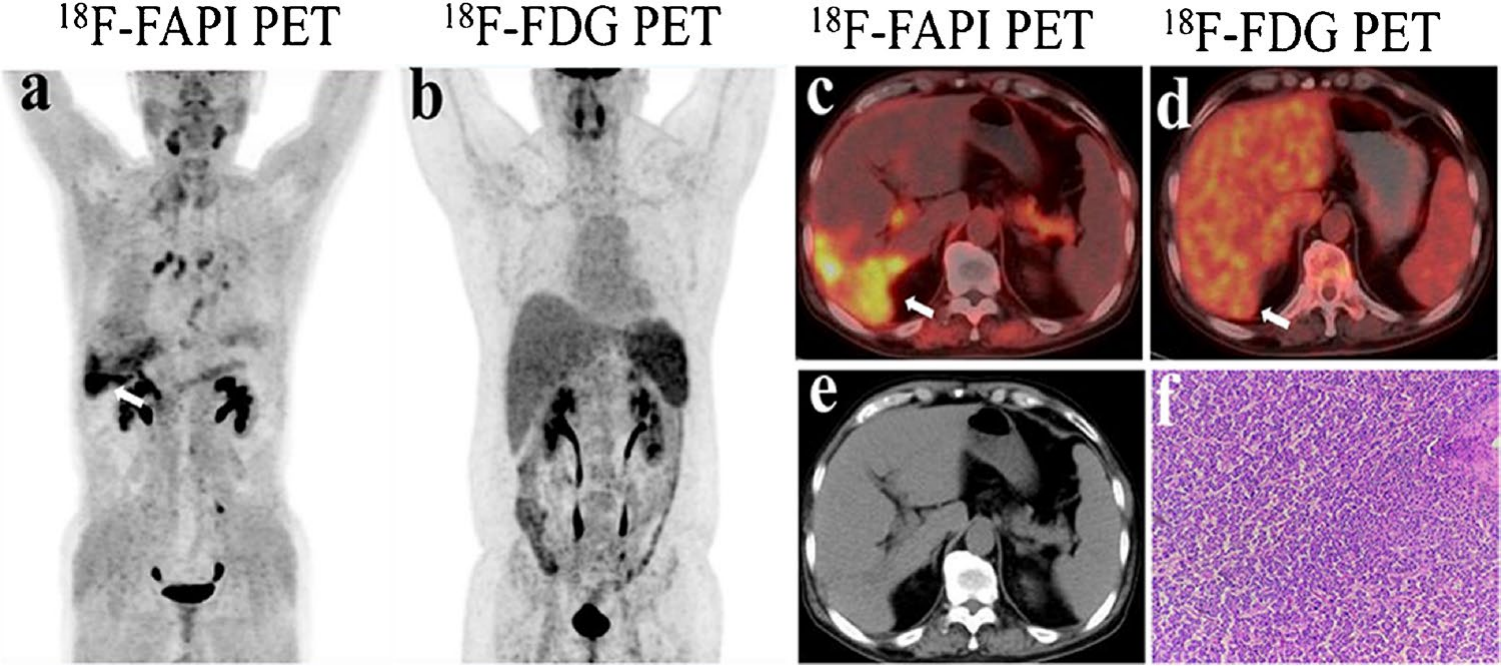
A 52-year-old male with liver discomfort underwent both ^18^F-FAPI-04 PET/CT and ^18^F-FDG PET/CT imaging for initial assessment. **a**, **c** ^18^F-FAPI-04 PET/CT images show low-to-moderate uptake in the right hepatic lesion (SUVmax = 8.73). **b**, **d** The paired ^18^F-FDG PET/CT images show negative findings in a patient with well-differentiated hepatocellular carcinoma. **e** The CT scan shows no abnormal nodule or mass in the corresponding region. **f** Liver biopsy from the FAPI-avid lesions helped to confirm the primary liver lesion

**Fig. 4:**
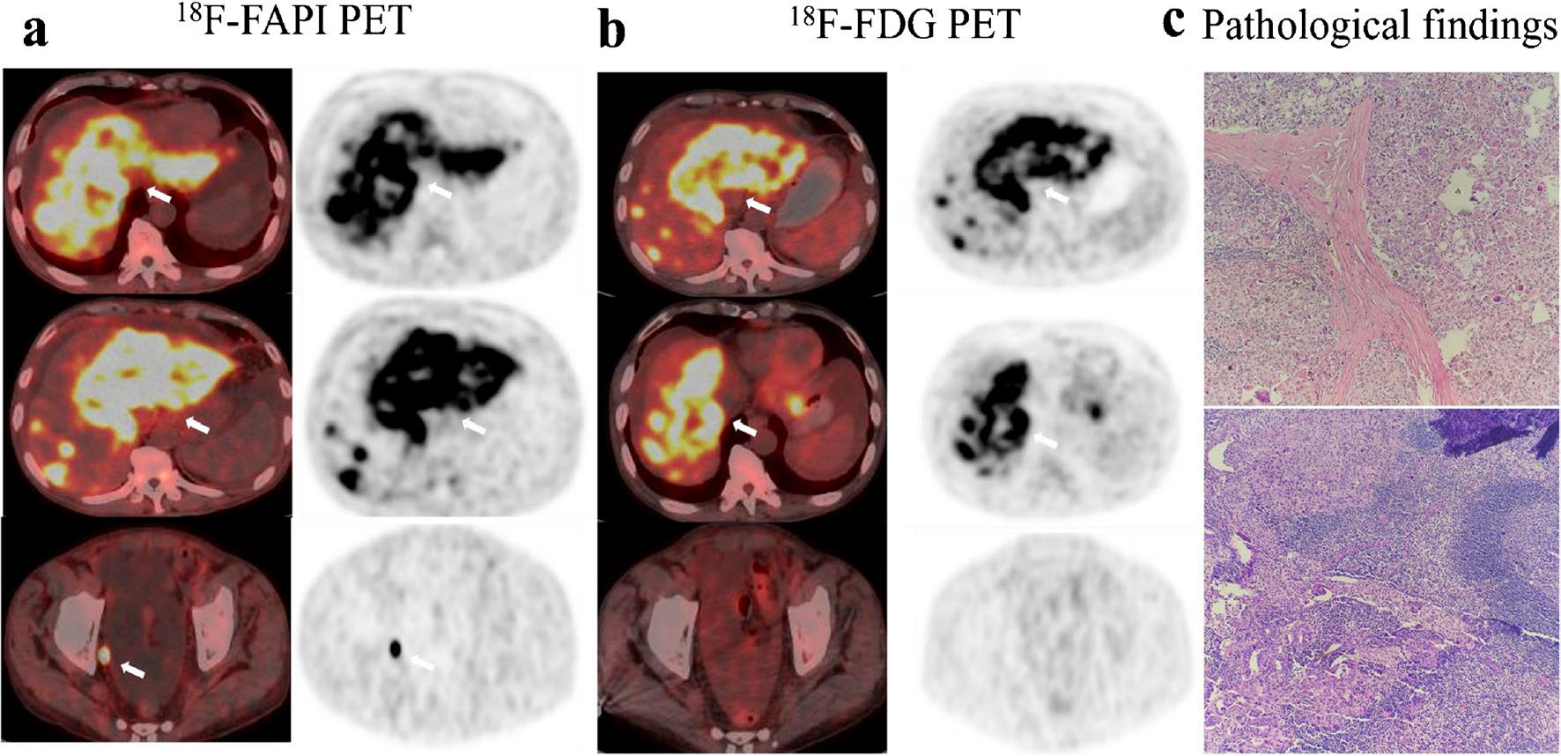
A 49-year-old male underwent PET/CT to evaluate a liver mass previously detected through ultrasound. **a**, **b** Intense metabolic activity was observed on ^18^F-FAPI-04 PET/CT images in the both primary lesion (SUVmax = 9.7) and lymph node (SUVmax = 3.3) (arrows). **c**, **d** The paired ^18^F-FDG PET/CT images also showed obvious uptake in the primary tumor (SUVmax = 6.9) but no uptake in the regional lymph node. **e** The pathological results derived from a liver mass revealed a primary poorly differentiated hepatocellular carcinoma. The pathological findings derived from lymph node resection are indicative of metastatic one

**Fig. 5:**
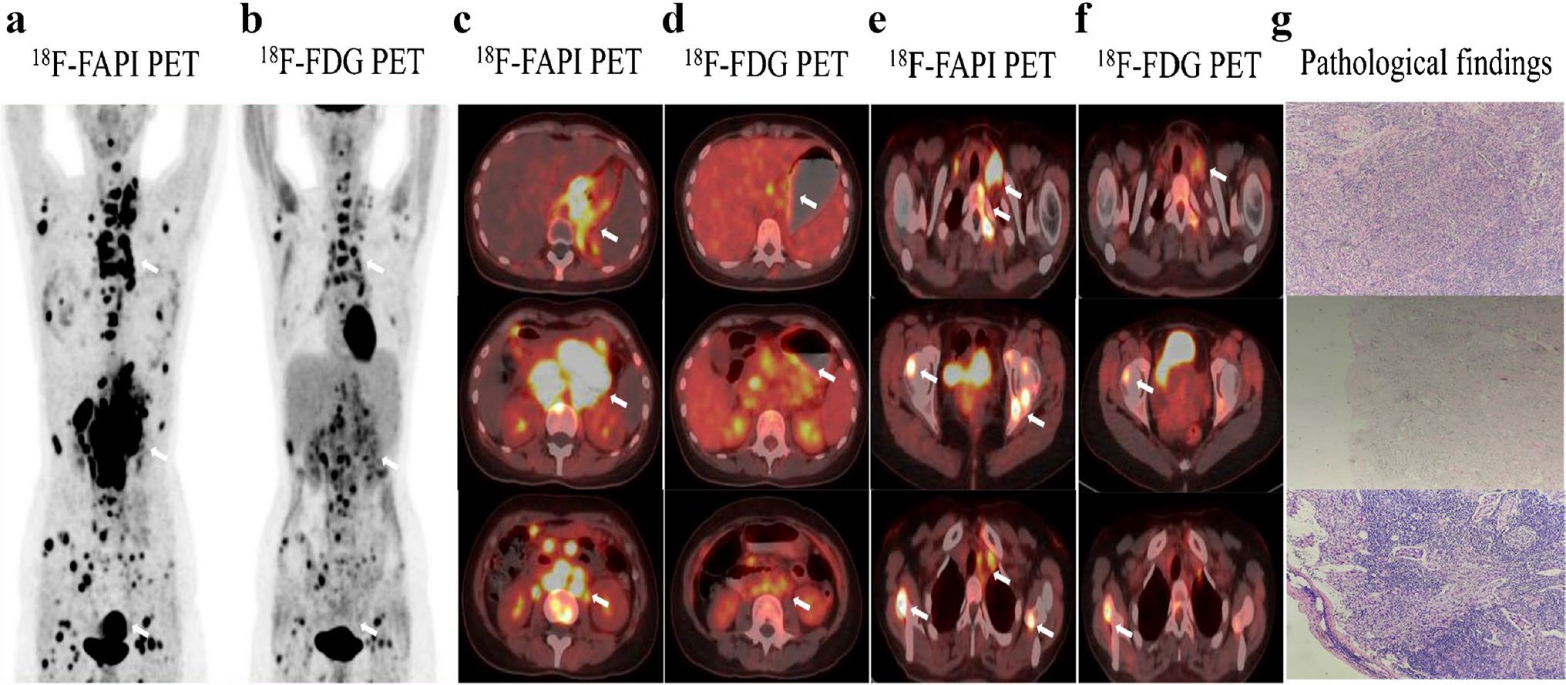
A 54-year-old male with gastric adenocarcinoma confirmed by pathological biopsy under gastroscopy. **a**, **c**, **e** ^18^F-FAPI-04 PET/CT displayed intensely diffuse uptake in the primary tumor and metastatic lesions (lymph node metastases, bone metastases and peritoneal metastases). **b**, **d**, **f** ^18^F-FDG PET/CT displayed slight uptake in the primary tumor but no uptake in several metastatic lesions. (g) The primary tumor and metastatic lesions were confirmed by the sequential pathological results

**Fig. 6:**
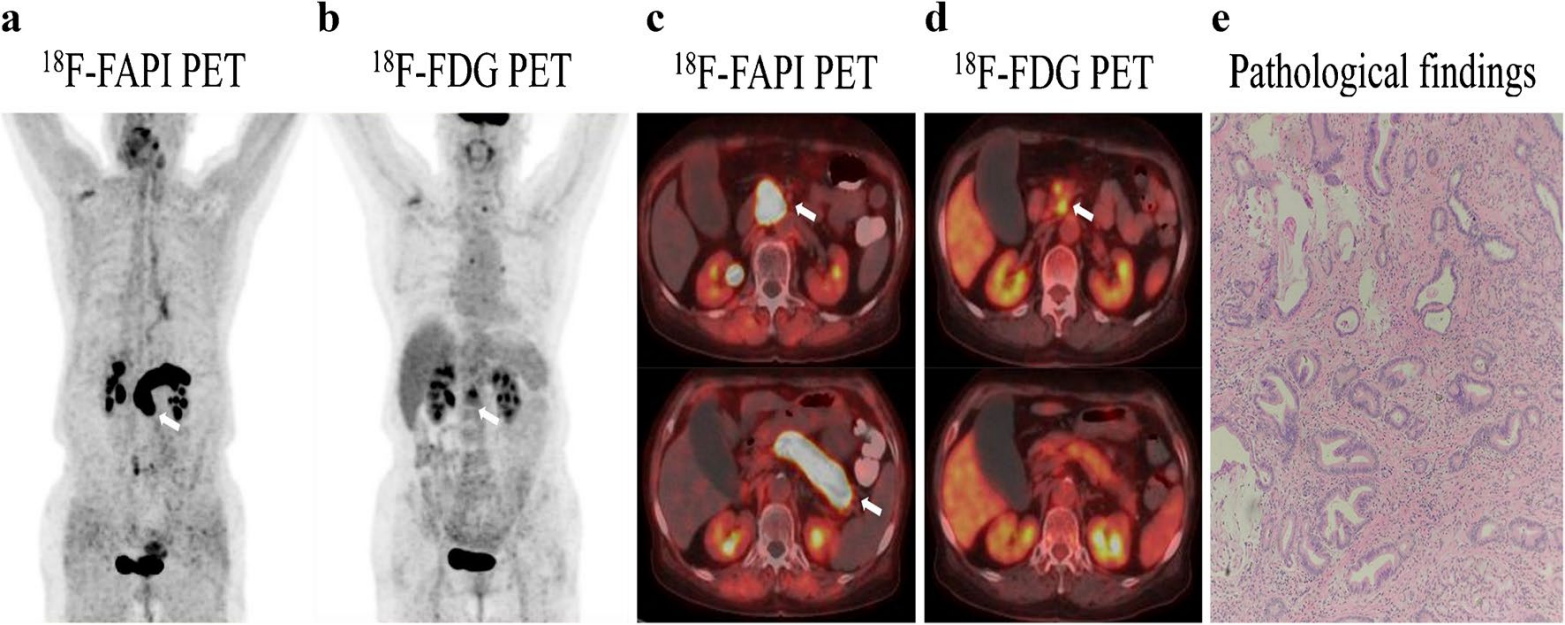
**a**, **c** Images from ^18^F-FAPI-04 PET/CT show an irregular lesion in the pancreas with intense metabolic activity, which strongly suggests pancreatic cancer. Intense and diffuse ^18^F-FAPI-04 uptake (SUVmax = 9.3) in the primary tumor and the body and tail of the pancreas was found. **b**, **d** Images from ^18^F-FDG PET/CT show two ill-defined nodules in the pancreas with low metabolic activity (SUVmax = 9.3). **e** The pathological findings derived from surgical resection are indicative of pancreatic cancer

Our findings have demonstrated that ^18^F-FAPI-04 has superiority over ^18^F-FDG mostly in the following cases: precise localization of the primary tumor and metastatic lesions, high activity retention with favorable contrast, high tumor-to-background ratio, accurate tumor staging, and potential impact on clinical management. Representative cases of the clinical application of this novel PET radiotracers were displayed in Figs. 1, 2, 3, 4, 5, 6 and Supplementary Figs. 2 to 5.

## Discussion

This prospective study aimed to compare the diagnostic efficacy of ^18^F-FDG PET/CT with ^18^F-FAPI-04 PET/CT in a limited cohort of patients with gastrointestinal system cancers. The present study found that ^18^F-FAPI-04 PET/CT demonstrated a significantly higher overall diagnostic efficacy than ^18^F-FDG PET/CT in patients with gastrointestinal malignancies. This efficacy was evident in the detection of more numerous or larger lesions, clarification of inconclusive findings from ^18^F-FDG PET, and the provision of valuable information for monitoring tumor recurrence. In comparison to ^18^F-FDG PET/CT, ^18^F-FAPI-04 PET/CT corrected the clinical stage in seven patients and necessitated a revised therapeutic regimen in three patients. The study thus provides substantial evidence that ^18^F-FAPI-04 PET/CT is a promising new imaging modality in the management of gastrointestinal cancer.

Prior studies have demonstrated the beneficial clinical utility of ^68^Ga-labeled FAPI-04 PET/CT imaging in diagnosing various cancer types. Specific to each cancer type investigated, Shi et al.'s study suggested that ^68^Ga-FAPI-04 PET/CT holds superior potential in detecting primary hepatic malignancies compared to ^18^F-FDG [10]. Lin et al. illustrated the great potential of ^68^Ga-FAPI-04 as a novel PET/CT tracer for detecting lymph nodes and distant metastases, consequently improving colorectal staging and prompting the optimization or alteration of treatment decisions [11]. Additionally, Pang et al. reported that ^68^Ga-FAPI-04 PET/CT exhibits greater sensitivity in detecting primary pancreatic tumors, involved lymph nodes, and distant metastases, compared with ^18^F-FDG PET/CT [12].

Regarding gastric cancer, FAPI PET/CT outperforms FDG PET/CT in detecting both primary gastric adenocarcinoma and peritoneal carcinomatosis originating from gastric cancer [13]. Existing findings indicate a significant limitation of ^18^F-FDG PET/CT in detecting gastric mucinous carcinoma and signet ring cell carcinoma. These specific pathological subtypes typically presented with small or diffuse growing patterns due to the scarcity of tumor cells, resulting in lower uptake of ^18^F-FDG, as the expression of tumor glucose transporter is relatively lower in these two histological types. Furthermore, the physiological ^18^F-FDG uptake of the gastric wall complicates tumor imaging. Conversely, the low background of ^18^F-FAPI-04 in the abdominopelvic cavity facilitates the application of ^18^F-FAPI-04 PET/CT in detecting gastric cancer [14]. Encouragingly, in our cohort of 14 patients with gastric cancer, all primary tumors were detected by ^18^F-FAPI-04 PET/CT (100%, 14/14), demonstrating a higher sensitivity than ^18^F-FDG PET/CT (71.42%, 10/14). Specifically, ^18^F-FAPI-04 PET/CT detected three cases of signet ring cell carcinoma of the stomach and one case of mucinous carcinoma that were overlooked by ^18^F-FDG PET/CT imaging. Consistent with previous studies focusing on ^68^Ga-labeled FAPIs, ^18^F-FAPI-04 PET/CT outperformed ^18^F-FDG PET/CT in terms of sensitivity, specificity, and accuracy for primary, nodal, and metastatic lesion characterization across different tumor types in our study. For instance, ^18^F-FAPI-04 PET/CT detected 97.7% (43/44) of the primary lesions with distinct tumor contours and demonstrated a higher TBR (7.9 vs. 2.4; *P* < 0.001) than ^18^F-FDG. In contrast, only 72.7% (32/44) of primary malignancies were identifiable on ^18^F-FDG PET/CT images. In semiquantitative analysis, the median SUV of ^18^F-FAPI-04 was more than double that of ^18^F-FDG. The uptake values in the majority of primary tumors in our study were comparable to those previously reported by Koerber et al [15].

Considering our patient-based findings, we caution against overestimating the role of FAPI in tumor imaging and diagnosis. This is because ^18^F-FAPI-04, while not a tumor-specific tracer, may accumulate in many non-oncological conditions, potentially resulting in false positives. In our study, false-positive uptake of ^18^F-FAPI-04 was observed in the following cases: 1) inflammatory diseases such as nasosinusitis and tumor-associated pancreatitis, 2) granulomatous diseases such as inflammatory granuloma, and 3) other conditions that induce fibrotic reactions, including those activated by radiation and surgery. We noted two instances where diffuse ^18^F-FAPI-04 uptake occurred due to tumor-associated pancreatitis, which could potentially cause an overestimation of tumor volume. Guided by prior research [16], we executed an additional 2-hour delayed ^18^F-FAPI-04 PET/CT scan to aid in differential diagnosis. The results from these dual-time scans showed differential kinetics between tumor-associated pancreatitis, which had decreased uptake, and pancreatic cancer, which demonstrated stable uptake. This observation underscores the importance of comprehensive image interpretation, emphasizing that it should not be solely dependent on the uptake level of ^18^F-FAPI-04. Instead, it must be combined with other imaging examinations, such as contrast-enhanced CT or MR scans, and supplemented with relevant clinical information.

As is well known, accurate lymph node staging is crucial for treatment decisions and it strongly influences the patients' survival prognosis [17]. The efficacy of ^18^F-FDG PET/CT in the context of gastrointestinal cancer with lymph node metastasis remains a contentious issue. Our data, however, demonstrated that ^18^F-FAPI-04 PET/CT was superior to ^18^F-FDG PET/CT in visualizing abdominal and pelvic lymph node metastases, corroborating findings from previous studies [8, 10]. The uptake of ^18^F-FAPI-04 in the positive lymph nodes was significantly higher than that of ^18^F-FDG. These findings underscore the potential of ^18^F-FAPI-04 PET/CT imaging to enhance the detection sensitivity of occult lymph node metastasis, thereby aiding clinicians in devising suitable treatment plans.

Gastrointestinal cancer is predisposed to hepatic metastasis. It is noteworthy, however, that ^18^F-FDG PET often yields false-negative results for small liver metastases. Our study showed that the sensitivity of ^18^F-FAPI-04 PET/CT was significantly higher than that of ^18^F-FDG PET/CT in detecting liver metastases, including small hepatic metastatic lesions with a diameter of less than 1 cm. Contrastingly, ^18^F-FAPI-04 PET/CT identified more suspected metastatic lesions in the liver than ^18^F-FDG PET/CT, a finding consistent with that of Deng et al. [18]. Peritoneal carcinomatosis is another common metastatic form in gastrointestinal cancer. Unfortunately, ^18^F-FDG PET occasionally displays a low sensitivity in visualizing peritoneal carcinomatosis, likely due to physiological radioactivity uptake in the intestinal tract. ^18^F-FAPI-04 PET/CT has been shown to be a promising imaging modality for the detection of peritoneal carcinomatosis, displaying a larger extent of the lesions [19]. In this study, we made the unexpected observation that ^18^F-FAPI-04 PET/CT yielded more positive findings in the peritoneum than ^18^F-FDG PET/CT. A similar observation was made by Zhao et al. [20], who reported a superior sensitivity of ^18^F-FAPI-04 PET/CT over ^18^F-FDG PET/CT for the detection of peritoneal carcinomatosis in patients with various cancer types. This advantage could potentially enhance image contrast and reduce the likelihood of missed diagnoses.

A critical clinical application of PET/CT is to evaluate the extent of disease involvement in recognized malignancies, both for staging and identifying tumor recurrence (restaging) [21]. Based on our results, ^18^F-FAPI-04 PET/CT appears to surpass ^18^F-FDG PET/CT in several key areas: 1) ^18^F-FAPI-04 PET/CT detected a larger number of involved lymph nodes than ^18^F-FDG PET/CT, demonstrating a higher sensitivity for the identification of metastatic lymph nodes; 2) ^18^F-FAPI-04 PET/CT surpassed ^18^F-FDG PET/CT in detecting liver, gastric, brain, lung, and bone metastases, even with small metastatic lesions (around 1cm in diameter). This is likely due to the markedly high uptake of ^18^F-FAPI-04 in metastases and relatively low background activity in these tissues; 3) ^18^F-FAPI-04 PET/CT outperformed ^18^F-FDG PET/CT in identifying peritoneal carcinoma. All peritoneal, omental, or mesenteric metastases were clearly depicted on ^18^F-FAPI-04 PET/CT with high contrast (SUV: median, 8.1; range, 4.9–12.1).

Overall, ^18^F-FAPI-04 PET/CT detected more positive lymph nodes and distant metastases than ^18^F-FDG, which resulted in alterations in TNM staging. It is worth noting that while ^18^F-FAPI-04 PET/CT demonstrated higher diagnostic accuracy than ^18^F-FDG PET/CT in this study, it did not exhibit particular advantages on patient management. Only a limited number of patients' treatment plans were adjusted based on ^18^F-FAPI-04 PET/CT findings. This may be because the same therapeutic regimen might be appropriate for patients at different clinical stages. Furthermore, for advanced patients (clinical stage IV), even if more lesions were identified by ^18^F-FAPI-04 PET/CT, the clinical stage remained the same, and the treatment plan was unchanged. However, it is important to highlight that treatment decisions were altered for half of the patients in the restaging group, indicating that ^18^F-FAPI-04 PET/CT plays a pivotal role in monitoring tumor recurrences and metastases, and in assessing therapeutic efficacy in treated gastrointestinal system cancer patients. Due to the limited number of patients in the restaging group, this observation warrants further validation with larger sample sizes.

Our study does have several limitations. Firstly, it is a preliminary report of an ongoing, single-center, prospective study on the diagnostic accuracy of ^18^F-FAPI-04 for PET/CT imaging of solid malignant tumors. The relatively small sample size may limit the power of the analysis. Hence, future prospective studies with larger patient populations are needed to further explore the role of ^18^F-FAPI-04 in cancer diagnosis and tumor imaging. Secondly, the sample was heterogeneous (comprising patients with gastric, pancreatic, liver, and colorectal cancers), and the limited number of patients with each cancer type precluded a subgroup analysis. As a result, we evaluated the primary and metastatic lesions of various cancer types collectively. Future research should strive to include larger numbers of patients with each specific cancer type to facilitate more rigorous statistical evaluations. Thirdly, although a prospective study was carried out, not all metastatic lesions could be biopsied due to technical and ethical considerations. Lastly, further characterization of FAP as a target using immunohistochemistry with anti-FAPα monoclonal antibodies should be conducted in future research.

Our findings suggest that ^18^F-FAPI-04 is a promising alternative to ^68^Ga-FAPI-04 and could potentially broaden the clinical application of FAPI PET/CT in tumor imaging. More specifically, ^18^F-FAPI-04 PET/CT exhibited higher tracer uptake and outperformed ^18^F-FDG PET/CT in detecting primary and metastatic lesions in patients with gastrointestinal system cancers. More importantly, ^18^F-FAPI-04 PET/CT prompted clinical management changes in over 20% of patients. Nonetheless, these results are only preliminary, and future multicenter research with larger sample sizes would provide a more comprehensive understanding of the clinical utility of ^18^F-FAPI-04 PET/CT in diagnosing gastrointestinal malignancies.

### Supplementary Information

Below is the link to the electronic supplementary material.

Supplementary file1 (PDF 1055 KB)

## Data Availability

The datasets generated during and analyzed during the current study are not publicly available due to patient privacy concerns but are available from the corresponding author on reasonable request.

## References

[1] Almuhaideb A, Papathanasiou N, Bomanji J (2011). ^18^F-FDG PET/CT imaging in oncology. Ann Saudi Med.

[2] Howard BA, Wong TZ (2021). ^18^F-FDG-PET/CT Imaging for Gastrointestinal Malignancies. Radiol Clin North Am.

[3] Kato H, Sohda M (2004). Kuwano H [Diagnosis of gastrointestinal tract malignancies using positron emission tomography (PET) with 18F-fluorodeoxyglucose (FDG)]. Nihon Shokakibyo Gakkai Zasshi.

[4] Liao Z, Tan ZW, Zhu P, Tan NS (2019). Cancer-associated fibroblasts in tumor microenvironment-Accomplices in tumor malignancy. Cell Immunol..

[5] Loktev A, Lindner T, Mier W, Debus J, Altmann A, Jager D (2018). A Tumor-Imaging Method Targeting Cancer-Associated Fibroblasts. J Nucl Med.

[6] Cermik TF, Ergul N, Yilmaz B, Mercanoglu G (2022). Tumor Imaging With ^68^Ga-DOTA-FAPI-04 PET/CT: Comparison With ^18^F-FDG PET/CT in 22 Different Cancer Types. Clin Nucl Med.

[7] Wang H, Zhu W, Ren S, Kong Y, Huang Q, Zhao J (2021). (^68^)Ga-FAPI-04 Versus (^18^)F-FDG PET/CT in the Detection of Hepatocellular Carcinoma. Front Oncol..

[8] Wei Y, Zheng J, Ma L, Liu X, Xu S, Wang S (2022). [(^18^)F]AlF-NOTA-FAPI-04: FAP-targeting specificity, biodistribution, and PET/CT imaging of various cancers. Eur J Nucl Med Mol Imaging.

[9] In H, Solsky I, Palis B, Langdon-Embry M, Ajani J, Sano T (2017). Validation of the 8th Edition of the AJCC TNM Staging System for Gastric Cancer using the National Cancer Database. Ann Surg Oncol.

[10] Shi X, Xing H, Yang X, Li F, Yao S, Congwei J (2021). Comparison of PET imaging of activated fibroblasts and ^18^F-FDG for diagnosis of primary hepatic tumors: a prospective pilot study. Eur J Nucl Med Mol Imaging.

[11] Lin X, Li Y, Wang S, Zhang Y, Chen X, Wei M (2023). Diagnostic value of [^68^Ga]Ga-FAPI-04 in patients with colorectal cancer in comparison with [^18^F]F-FDG PET/CT. Front Oncol.

[12] Pang Y, Zhao L, Shang Q, Meng T, Zhao L, Feng L (2022). Positron emission tomography and computed tomography with [^68^Ga]Ga-fibroblast activation protein inhibitors improves tumor detection and staging in patients with pancreatic cancer. Eur J Nucl Med Mol Imaging.

[13] Kuten J, Levine C, Shamni O, Pelles S, Wolf I, Lahat G (2022). Head-to-head comparison of [^68^Ga]Ga-FAPI-04 and [^18^F]-FDG PET/CT in evaluating the extent of disease in gastric adenocarcinoma. Eur J Nucl Med Mol Imaging.

[14] Jiang D, Chen X, You Z, Wang H, Zhang X, Li X (2022). Comparison of [^68^Ga]Ga-FAPI-04 and [^18^F]-FDG for the detection of primary and metastatic lesions in patients with gastric cancer: a bicentric retrospective study. Eur J Nucl Med Mol Imaging.

[15] Koerber SA, Staudinger F, Kratochwil C, Adeberg S, Haefner MF, Ungerechts G (2020). The Role of ^68^Ga-FAPI PET/CT for Patients with Malignancies of the Lower Gastrointestinal Tract: First Clinical Experience. J Nucl Med.

[16] Zhang H, An J, Wu P, Zhang C, Zhao Y, Tan D (2022). The Application of [^68^Ga]-Labeled FAPI-04 PET/CT for Targeting and Early Detection of Pancreatic Carcinoma in Patient-Derived Orthotopic Xenograft Models. Contrast Media Mol Imaging.

[17] Ott K, Blank S, Ruspi L, Bauer M, Sisic L, Schmidt T (2017). Prognostic impact of nodal status and therapeutic implications. Transl Gastroenterol Hepatol.

[18] Deng M, Chen Y, Cai L (2021). Comparison of ^68^Ga-FAPI and ^18^F-FDG PET/CT in the Imaging of Pancreatic Cancer With Liver Metastases. Clin Nucl Med.

[19] Güzel Y, Kaplan İ (2023). Comparison of ^68^GA-FAPI-04 PET/CT and ^18^F-FDG PET/CT findings in peritonitis carcinomatosa cases. Hell J Nucl Med.

[20] Zhao L, Pang Y, Luo Z, Fu K, Yang T, Zhao L (2021). Role of [^68^Ga]Ga-DOTA-FAPI-04 PET/CT in the evaluation of peritoneal carcinomatosis and comparison with [^18^F]-FDG PET/CT. Eur J Nucl Med Mol Imaging.

[21] Lightdale CJ (1996). Diagnosis, staging, and cure of early gastrointestinal cancer. Gastrointest Endosc.

