# Supplementary material for: [18F] AlF‑NOTA‑FAPI‑04 PET/CT as a promising tool for imaging fibroblast activation protein in gastrointestinal system cancers: a prospective investigation of comparative analysis with 18F-FDG
Source: Eur J Nucl Med Mol Imaging. 2023 Aug 5;50(13):4051–63. doi: 10.1007/s00259-023-06351-9 (PMC10611594; doi:10.1007/s00259-023-06351-9)
Supplement: Supplementary file 1 — Supplementary file1 (PDF 1055 KB) [file 259_2023_6351_MOESM1_ESM.pdf]

Supplementary Materials

Supplementary data 1

Preparation of [^{18}F] AlF-NOTA-FAPI-04:

1. Reagent preparation

1.1 Preparation of buffer solution Take 0.9 g NaAc, dissolve it into 100 ml of water for injection, measure the pH value of the solution with a pH meter, and add HAc into the solution until the pH value drops to 4.0. The volume of HAc used is about 1.2 ml.

1.2 Preparation of aluminum trichloride solution Weigh 0.2415 g of AlCl3.6H2O and dissolve it in 100 ml of injection water to prepare 10 mmol/L aluminum trichloride solution.

**Reagent name**	**supplier**	**specifications**	**Item No.**
NOTA-FAPI-04	Nanchang Probe Biotechnology Co., Ltd	5mg/bottle	
Sodium acetate	Chinese medicine reagent	500g/ bottle	CAS127-09-3 V900212
Glacial acetic acid	SIGMA	100ml/ bottle	A6283-100ML
Aluminium trichloride	SIGMA	5g/ bottle	56319-5G
Anhydrous acetonitrile	SIGMA	100ml/ bottle	acetonitrile

2. Preparation of precursor solution

2.1 Weigh 1mg of NOTA-FAPI precursor and put it into a vial, use a pipette gun to extract 500 uL of acetonitrile and 500 uL of 1.1 buffer, and add the vial to dissolve the precursor.

2.2 Use a pipette gun to weigh 200 uL of 2.1 precursor solution and add it into a 3 ml penicillin bottle; Then add 10 uL aluminum trichloride solution into it and mark it as #3 bottle.

2.3 Add 300 uL acetonitrile and 225 uL buffer solution to #3 bottle.

3. Preparation of other reagents

1 # bottle 0.3 ml physiological saline + 0.3 ethanol.

2 # bottle 0.5 ml acetonitrile.

3 # bottle 2.3.

4 # bottle 8 ml physiological saline.

5 # bottle 8 ml physiological saline.

6 # bottle 2 ml of 50% ethanol solution.

4. Synthesis steps

4.1 The accelerator bombards the heavy oxygen water to obtain $^{18}F^-$, which is transferred to the CFN200 synthesis module.

4.2 Capture the QMA column and record the activity of RI1.

4.3 0.6 ml physiological saline from 1 # bottle is used to wash the QMA column into the reaction tube and heat it for the first time.

4.4 Add 0.5 ml acetonitrile from #2 bottle to the reaction tube and heat it for secondary azeotropic water removal.

4.5 The precursor of #3 bottle is added to the reaction tube and heated at 105 ℃ for 12 minutes.

4.6 Add #4 bottle of normal saline into the reaction tube, dissolve the reaction solution, and then mount the C-18 column to capture the product.

4.7 Clean the reaction tube and C-18 column with #5 normal saline.

4.8 #6 clean C-18 column and filter the membrane to get the product.

5. Quality control

0.25% methanol of pump A contains 0.08% trifluoroacetic acid; 0.75% water for pump B; Flow rate 1ml/min column temperature=room temperature; Retention time 5.15 min.

^{18}F-AlF-NOTA-FAPI-04 Injection Quality Inspection Report:

[Character] Colorless clear solution meets the requirements

[Identification] Positron nuclides 0.511 MeV and 1.022 MeV meet the requirements

[pH value] 5.0 ~ 8.0

[Bacterial endotoxin] less than 15 EU/mL less than 15 EU/mL

[Sterile] No bacteria, mold detection meets the requirements

[Radiochemical purity] > 90% ~ 100%

[Radioactive concentration] is greater than 370 MBq/mL, meeting the requirements

[Radio nuclear purity] > 95% ~ 99%

[Stability at room temperature] The radiochemical purity is more than 90% (48 hours), meeting the requirements

Conclusion: The quality standard test results of ^{18}F-FDG injection of this product (the second part of the Chinese Pharmacopoeia, 2015 edition) meet the requirements

Reference

1. Wei Y, Zheng J, Ma L et al. [^{18}F] AlF-NOTA-FAPI-04: FAP-targeting specificity, biodistribution, and PET/CT imaging of various cancers. Eur J Nucl Med Mol Imaging. 2022 Jul;49(8):2761-2773. doi: 10.1007/s00259-022-05758-0. Epub 2022 Mar 9. PMID: 35262766.

Supplementary data 2

Supplementary Figure 1

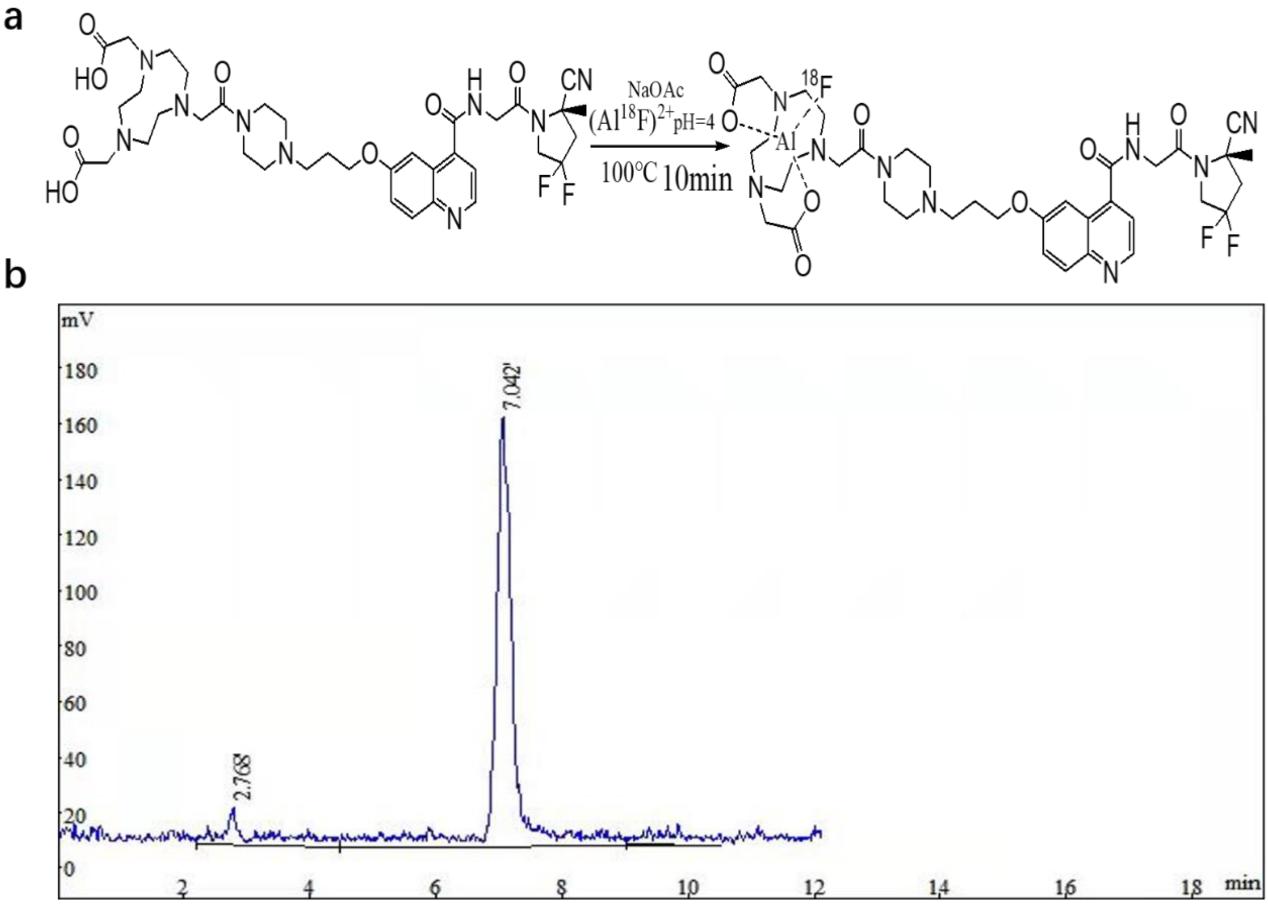

Supplementary Figure 1. [^{18}F] labeled chemical structural formula of AlF-NOTA-FAPI-04 (a). Radioactive high performance liquid chromatography (HPLC)[^{18}F] AlF-NOTA-FAPI-04 (b).

Supplementary Figure 2

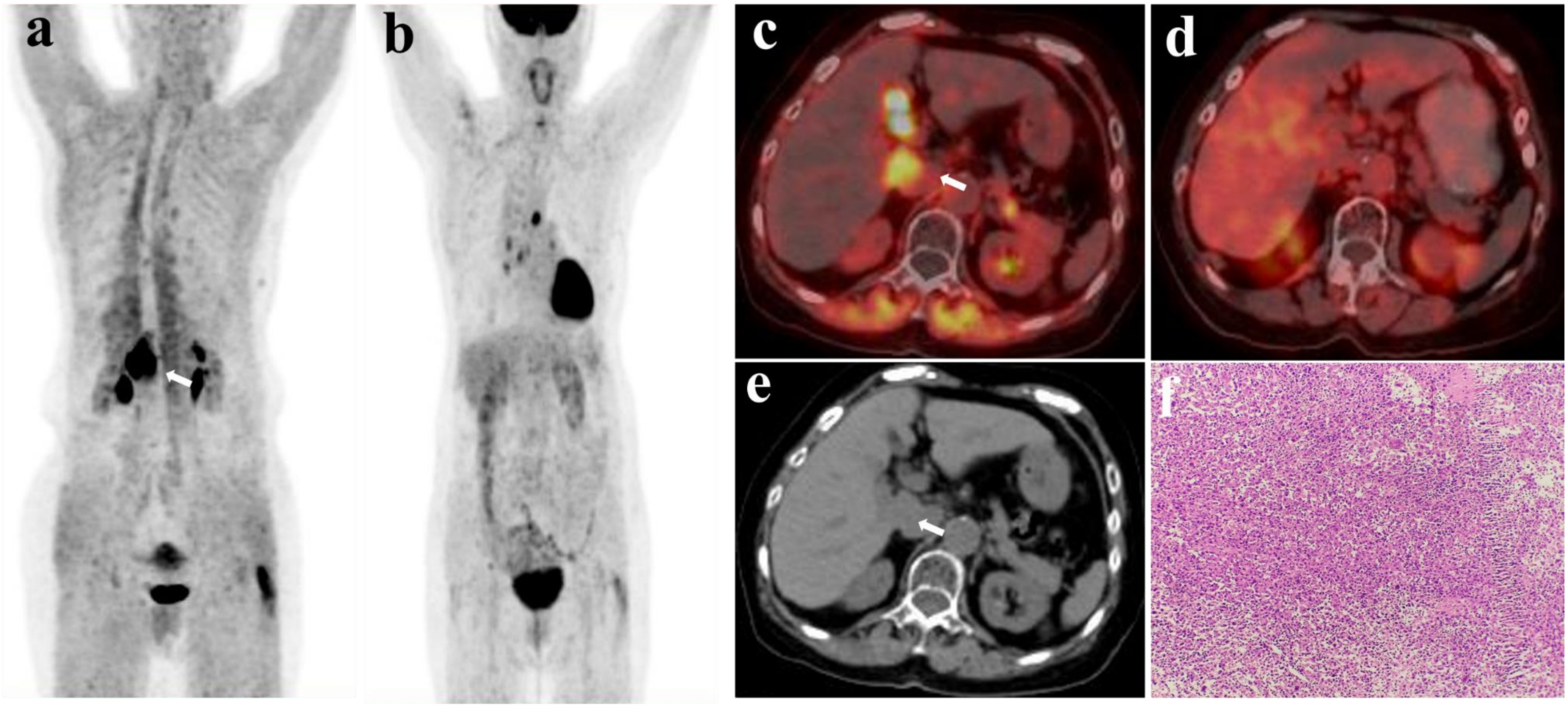

Supplementary Figure 2. A 61-year-old female with liver cancer who has been treated with radiotherapy and chemotherapy, whose tumor markers continue to increase recently, and tumor local recurrence was considered. This patient underwent PET/CT for tumor restaging before treatment. ^{18}F-FAPI-04 PET/CT (a, c) showed a lesion near the caudate lobe (arrows). The CT scan shows a slightly lower density in the corresponding region (e). Liver biopsy from the FAPI-avid lesions helped to confirm the recurrent liver lesion (f).

Supplementary Figure 3

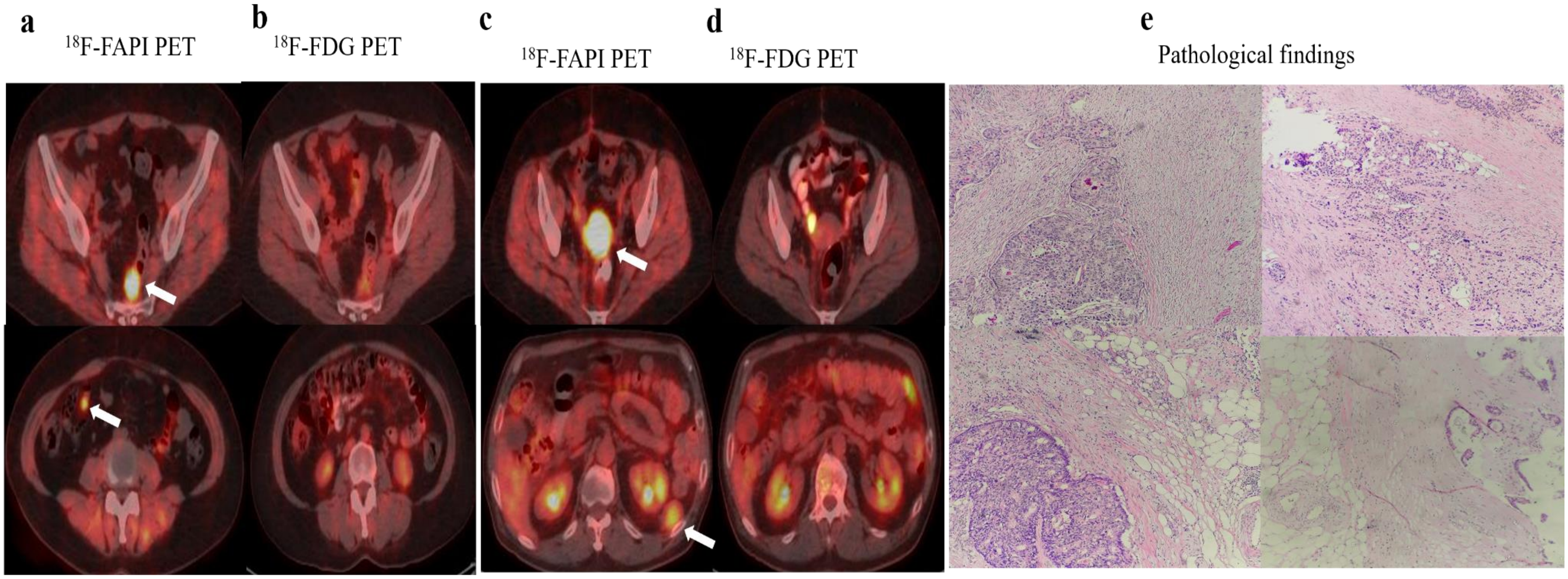

Supplementary Figure 3. Two patients who have undergone surgical resection of rectal cancer underwent both ^{18}F-FAPI-04 PET/CT and ^{18}F-FDG PET/CT imaging for tumor restaging. (a, c) ^{18}F-FAPI-04 PET/CT images revealed a nodule or a mass lesion in the original surgical area and tumor recurrence was considered. In addition, ^{18}F-FAPI-04 PET/CT images showed high uptake in the peritoneum. (b, d) ^{18}F-FDG PET/CT showed negative uptake in the corresponding (SUVmax = 2.8) but negative uptake in tumor area and peritoneum. (e) Rectal biopsy at the primary lesion confirmed the tumor recurrence of moderately differentiated and poorly differentiated rectal cancer, and the corresponding peritoneal metastasis was also validated.

Supplementary Figure 4

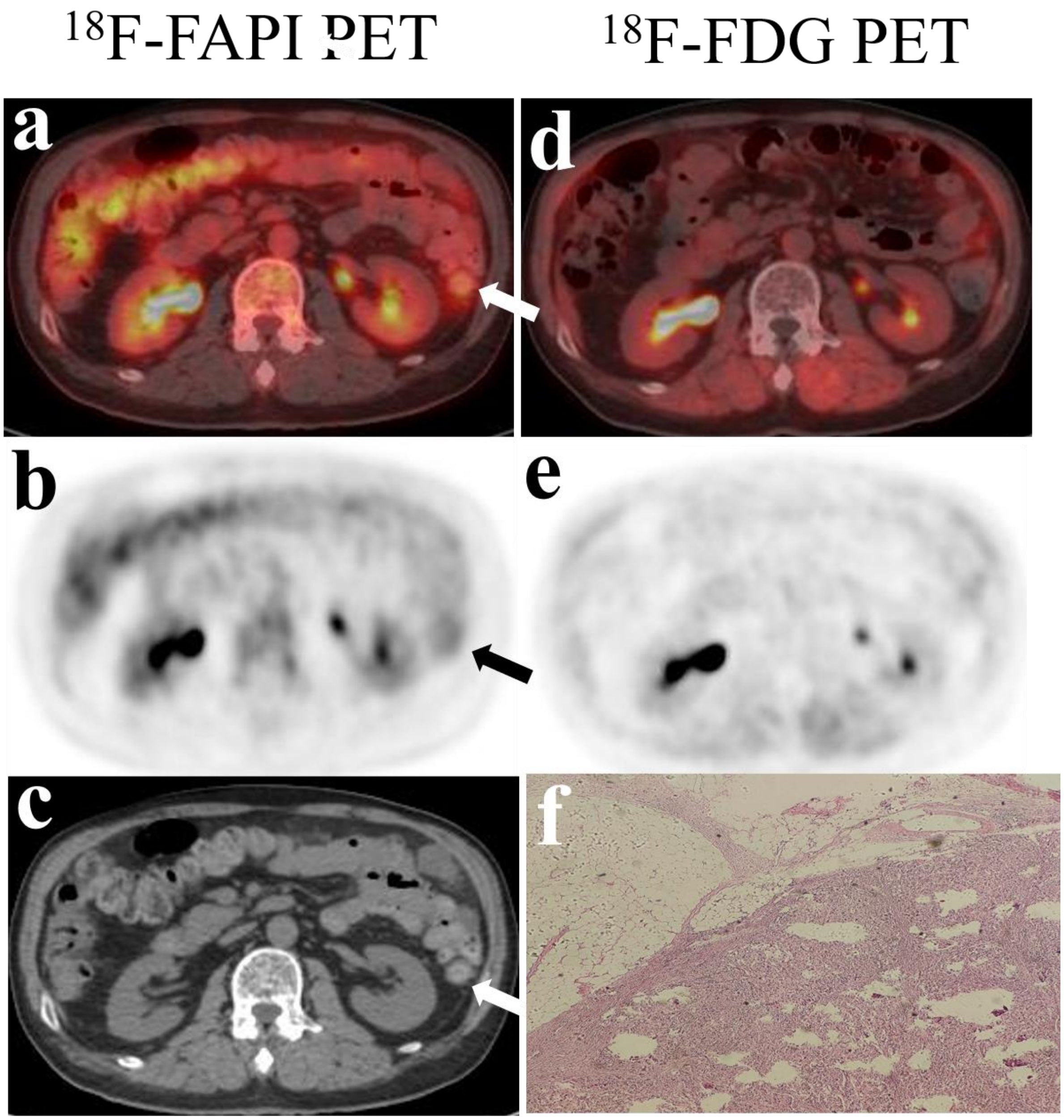

Supplementary Figure 4. A 61-year-old male who have undergone surgical resection of rectal cancer 1 year prior underwent both ^{18}F-FAPI-04 PET/CT and ^{18}F-FDG PET/CT imaging for tumor restaging. (a) ^{18}F-FAPI-04 PET/CT images revealed obvious uptake in the left pulmonary hilum lymph node on both maximum-intensity-projection (MIP) and axial images (SUVmax=6.2), but negative uptake was observed on ^{18}F-FDG PET/CT(b). The lesion was ultimately confirmed as inflammatory hyperplasia of lymph node by needle biopsy.

Supplementary Figure 5

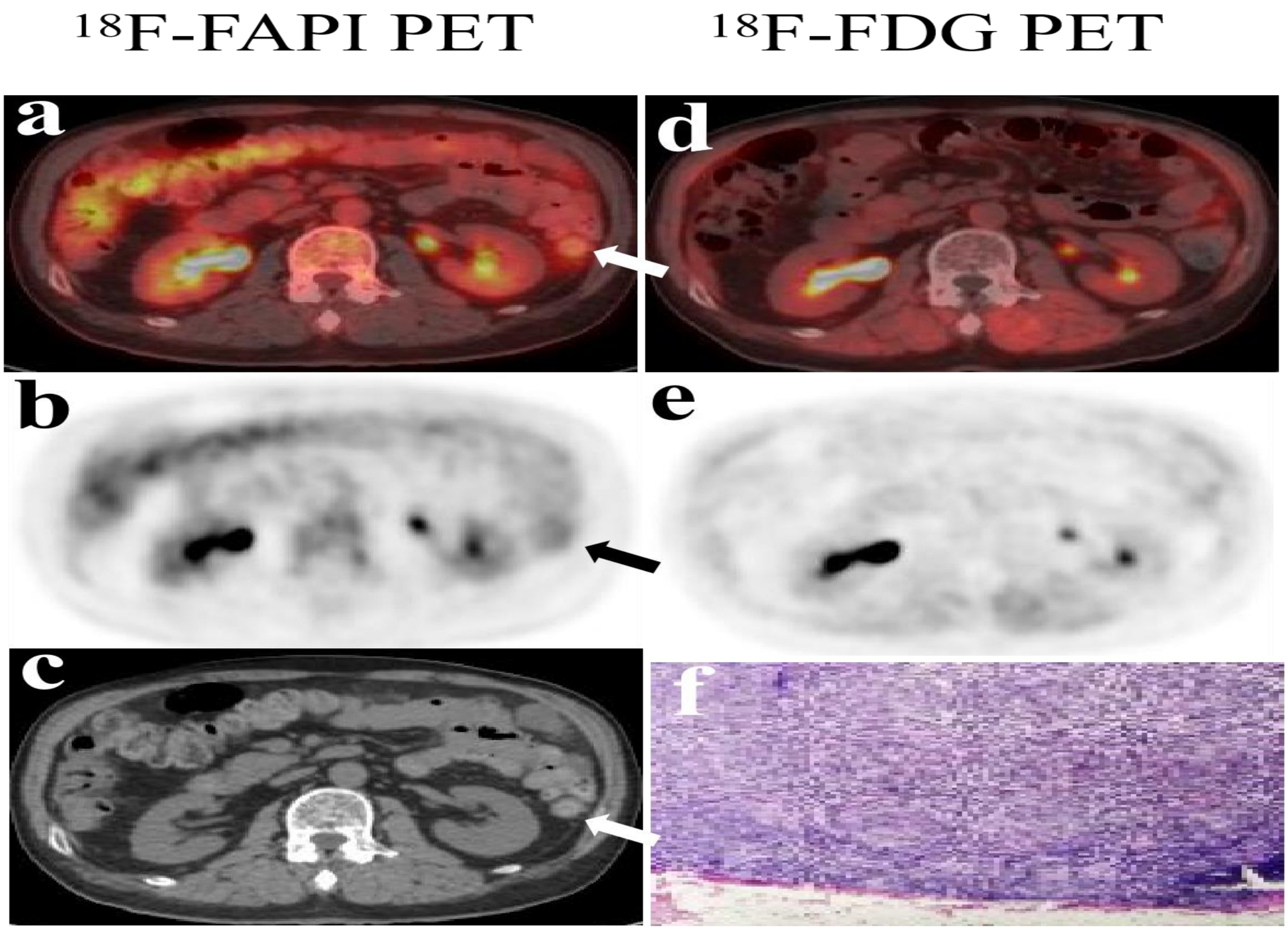

Supplementary Figure 4. Figure 4. A 70-year-old male who have undergone surgery, chemotherapy, and radiotherapy of rectal cancer underwent both ^{18}F-FAPI-04 PET/CT and ^{18}F-FDG PET/CT imaging for tumor restaging. (a, b) ^{18}F-FDG PET/CT images revealed low-to-moderate uptake in the peritoneum on both maximum-intensity-projection (MIP) and axial images (SUVmax=3.2), while the CT scan shows an obvious nodule in the corresponding region (c). The paired ^{18}F-FAPI-04 PET/CT reveals no abnormal activity on the MIP (d) and axial images (e). Although no significantly intense metabolic activity was observed on^{18}F-FDG PET/CT, peritoneal metastasis was considered due to persistently elevated tumor markers. (f) The pathological results derived from a needle biopsy validated our conclusion.

Supplementary data 3

Supplementary Table 1

Table S1 Therapeutic regimen of reference standard and that prompted by FAPI or FDG PET-CT imaging.

Management implication	Reference standard	Actual same as the reference standard	FAPI-04	Actual same as FAPI	FDG	Actual same as FDG
Initial staging group **(No.)**	44		44		44	
No treatment needed/ follow-up	0	0	0	0	0	0
Surgical resection	13	12	13	13	8	7
Perioperative chemotherapy/ radio-chemotherapy plus surgical resection	20	20	17	18	24	22
Non-surgical candidate	11	9	14	8	12	9
Restaging group **(No.)**	16		16		16	
Maintenance of previous treatment	6	4	4	5	12	5
Modification of previous treatment	10	8	12	7	14	3
